# Impact of Various Essential Oils on the Development of Pathogens of the *Fusarium* Genus and on Health and Germination Parameters of Winter Wheat and Maize

**DOI:** 10.3390/molecules29102376

**Published:** 2024-05-18

**Authors:** Jakub Danielewicz, Monika Grzanka, Łukasz Sobiech, Ewa Jajor, Joanna Horoszkiewicz, Marek Korbas, Andrzej Blecharczyk, Kinga Stuper-Szablewska, Kinga Matysiak

**Affiliations:** 1 Department of Mycology, Institute of Plant Protection, National Research Institute, Władysława Wegorka 20, 60-318 Poznan, Poland; j.danielewicz@iorpib.poznan.pl (J.D.); e.jajor@iorpib.poznan.pl (E.J.); j.horoszkiewicz@iorpib.poznan.pl (J.H.); m.korbas@iorpib.poznan.pl (M.K.); 2 Department of Agronomy, Faculty of Agronomy, Horticulture and Biotechnology, Poznań University of Life Sciences, Wojska Polskiego 28, 60-637 Poznan, Poland; monika.grzanka@up.poznan.pl (M.G.); andrzej.blecharczyk@up.poznan.pl (A.B.); 3 Department of Chemistry, Faculty of Forestry and Wood Technology, Poznań University of Life Sciences, Wojska Polskiego 28, 60-637 Poznań, Poland; kinga.stuper@up.poznan.pl; 4 Department of Herbology and Plant Protection Technology, Institute of Plant Protection, National Research Institute, Władysława Wegorka 20, 60-318 Poznan, Poland; k.matysiak@iorpib.poznan.pl

**Keywords:** biofungicides, seedling blight, plant protection

## Abstract

Currently, researchers are looking for ways to replace synthetic pesticides with substances of natural origin. Essential oils are produced by plants, among other things, to protect against pathogens, which is why there is interest in their use as fungicides. This experiment assessed the composition of essential oils from a commercial source, their impact on the development of mycelium of pathogens of the *Fusarium* genus, and the possibility of using them as a pre-sowing treatment. Grains of winter wheat (*Triticum aestivum* L.) and corn (*Zea mays* L.) were inoculated with a suspension of mycelium and spores of fungi of the *Fusarium* genus and then soaked in solutions containing oils of sage (*Salvia officinalis* L.), cypress (*Cupressus sempervirens* L.), cumin (*Cuminum cyminum* L.), and thyme (*Thymus vulgaris* L.). The obtained results indicate that thyme essential oil had the strongest effect on limiting the development of *Fusarium* pathogens and seedling infection, but at the same time it had an adverse effect on the level of germination and seedling development of the tested plants. The remaining essential oils influenced the mentioned parameters to varying degrees. Selected essential oils can be an alternative to synthetic fungicides, but they must be selected appropriately.

## 1. Introduction

Maize and wheat are among the most important crops in the world [1]. Their cultivation plays a key role in maintaining global food security [2]. Many factors influence the yield levels of the abovementioned cereals. These include weather conditions, soil properties, and the occurrence of weeds and pests [3,4]. The volume and quality of harvested crops are also influenced by the occurrence of plant diseases [5,6].

Some of the most important pathogens occurring in the cultivation of maize and wheat are fungi of the genus *Fusarium* [7]. They infect plants at various stages of development and contribute to a decrease in yield, and some of them are able to contaminate the grain with mycotoxins [8,9]. The first treatment that helps protect plants against diseases is seed dressing [10]. Seed treatments should be selected not only for their efficacy in controlling diseases but also for the safety of protected crops, to avoid the risk of phytotoxicity, to limit seed germination and seedling emergence, and to be safe for the natural environment [11]. Application of seed treatment is important due to the fact that infection of seedlings may occur, among other factors, as a result of infection from the soil or with seed material [12]. Fungi of the *Fusarium* genus can also overwinter in the form of mycelium or chlamydospores in crop residues and then infect plants [13]. One of the most important diseases caused by *Fusarium* is seedling blight [14]. In the case of pre-emergence blight, infected sprouts darken and rot and do not reach the soil surface. The occurrence of post-emergence blight is visible by infection of the subcotyledon part of the root system and watery, brown spots visible on the stems, which may result in the death of the seedlings [15].

*Fusarium avenaceum* can produce mycotoxins—moniliformin (MON), beauvericin (BEA), and enniatins (ENNs) [16]. The teleomorph stage of *Gibberella avenacea* is rarely seen [17]. Macroconidia of *Fusarium avenaceum* are long, -walled, and narrow, while microconidia are rare [18]. Mycotoxins produced by *Fusarium culmorum* are nivalenol (NIV), deoxynivalenol (DON), and 3-acetyl-deoxynivalenol (3-ADON) [19]. The teleomorph of this species is unknown [20]. *Fusarium culmorum* has short, curved, thick-walled macroconidia [21]. *Fusarium graminearum* produces type B trichothecene (TCTB) mycotoxins [22]. The teleomorph of this species is *Gibberella zeae* [23]. Macroconidia of this species are straight or slightly curved [24]. *Fusarium fujikuroi* can produce moniliformin (MON), fumonisin B1 (FB1), and beauvericin (BEA) [25]. The teleomorph is *Gibberella fujikuroi* [26]. The macroconidia of *Fusarium fujikuroi* are slender [27]. All mentioned fungal species can cause seedling blight [28,29,30,31].

Essential oils are substances that plants produce to protect against pests, pathogens, and water loss and to attract pollinating insects [32,33]. They may also have allelopathic effects [34]. They are mixtures of various chemical substances, especially terpenes, ketones, aldehydes, esters, and lactones, produced in the secretory tissues of various parts of plants [35,36]. The essential oil of sage (*Salvia officinalis* L.) is used by human beings, e.g., for its antiseptic and anti-inflammatory effects [37]. Oil produced from cypress (*Cupressus sempervirens* L.) has anticancer, antiparasitic, and estrogenic properties [38]. Cumin (*Cuminum cyminum* L.) is another plant that produces essential oils. Its oils have, among others, antioxidant and antimicrobial properties [39]. The essential oil produced by thyme (*Thymus vulgaris* L.) has antiseptic, antiviral, and bactericidal properties [40]. Currently, research is underway on the possibility of using essential oils to protect plants during their development and storage [41,42,43]. Research is underway on the possibility of using them as natural fungicides, herbicides, and insecticides [44]. There are examples of plant-protection products based on essential oils that have been introduced to the market [45]. It is well-known fact that essential oils are mixtures of several compounds [46]. The application of a preparation containing various substances with different effects gives hope for reducing the risk of resistance [47]. The search for natural plant-protection options that will be alternatives to chemical pesticides is related, among other things, to the approach to consumer health [48]. Pesticides could have a wide range of effects on non-targeted organisms, aquatic ecosystems, and plant physiology, resulting in environmental issues [49]. Currently, synthetic fungicides are commonly used to treat grain; one example of such a fungicide is a.i. prothioconazole. This substance belongs to the group of triazoles [50]. Attention is drawn to the possibility of introducing essential oils for plant protection due to the need to reduce the occurrence of pesticide residues [51]. The factor that makes their application difficult is the fact that essential oils are difficult to dissolve in water. Therefore, various formulations are being developed to enable their use in agriculture [52].

The aim of this study was to assess the impact of essential oils from sage, evergreen cypress, cumin, and thyme on the development and health of winter wheat and maize seedlings.

## 2. Results

The composition of essential oils depended on the plant from which the compounds came (Table 1). The composition of sage essential oil was dominated by α-Thujone, and the second most important ingredient was camphor. In the case of thyme essential oil, the dominant substances were trans-Thujanol and α-Thujene. ß-Pinene and myrcene were the main components of cumin essential oil, while α-Pinene and δ-3-carene dominated in the composition of cypress essential oil.

**Figure 1:**
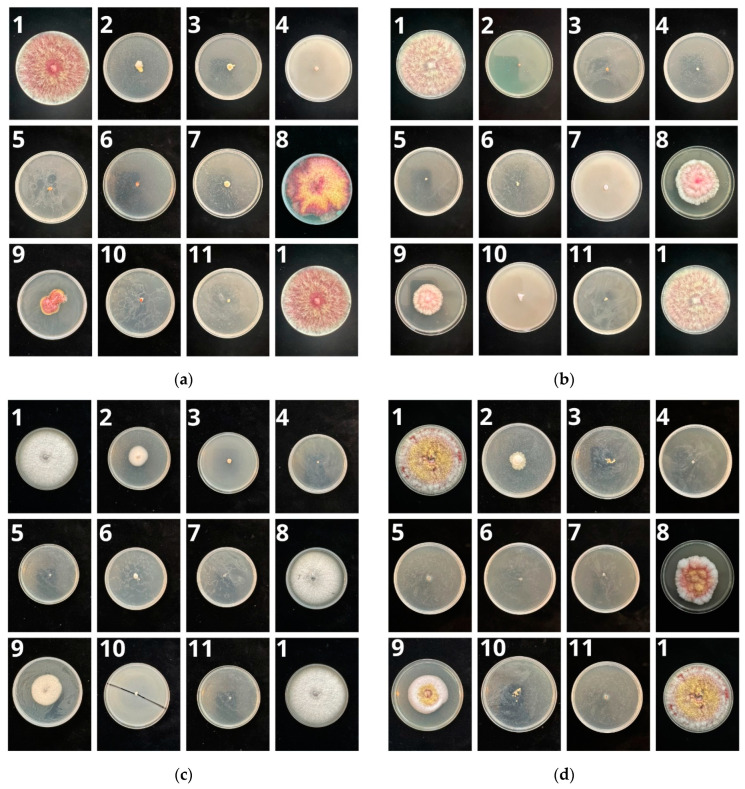
The influence of selected essential oils and prothioconazole on the growth of mycelium of pathogens of the genus *Fusarium*: (**a**) *F. culmorum*; (**b**) *F. graminearum*; (**c**) *F. fujikuori*; (**d**) *F. avenaceum*. The photo numbers correspond to the numbering in Table 2.

The level of inhibition of the development of individual fungi of the *Fusarium* genus depended on the substance used (Table 2, Figure 1). In the case of *F. culmorum*, *F. fujikuori*, and *F. avenaceum*, the strongest inhibition of mycelium development was observed for sage essential oil (EO) used at a higher dose, thyme EO, cumin EO, and a.i. prothioconazole. In the case of *F. graminearum*, no differences were found between sage EO doses. In all cases, cypress essential oil had the lowest level of influence on the mycelium development of the tested pathogens.

Most substances had no effect on the energy and germination capacity of wheat and corn grains (Table 3). In the case of the first of the tested cereal species, a decrease in the values of the discussed parameters was found for both doses of thyme EO; for corn, negative effects on germination were noted only for the higher dose of the mentioned essential oil.

**Figure 2:**
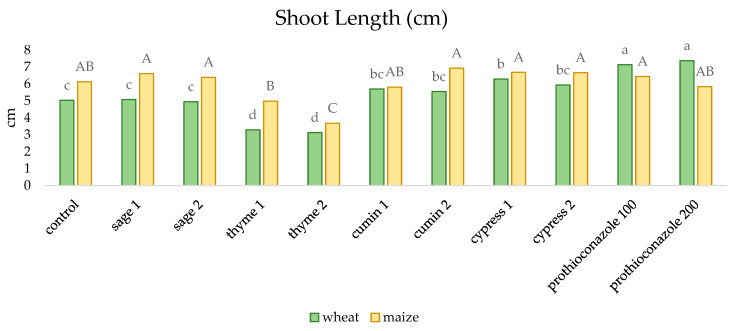
The effect of essential oils and prothioconazole on the length of winter wheat and maize shoots (numerical values given in the names of the substances correspond to doses (mL) per 200 mL of water or 100 kg of grain). Different letters indicate statistics different mean HSD (0.05): wheat = 0.815 (lower-case letters), SD = 0.57; maize = 0.917 (capital letters), SD = 0.64.

Individual substances had different effects on the shoot lengths of winter wheat and maize (Figure 2). In the case of corn, significantly shorter shoots were observed only when thyme essential oil was used. The longest shoots of winter wheat were recorded for the combination in which prothioconazole was used, while the shortest shoots were recorded for grains soaked in thyme EO.

**Figure 3:**
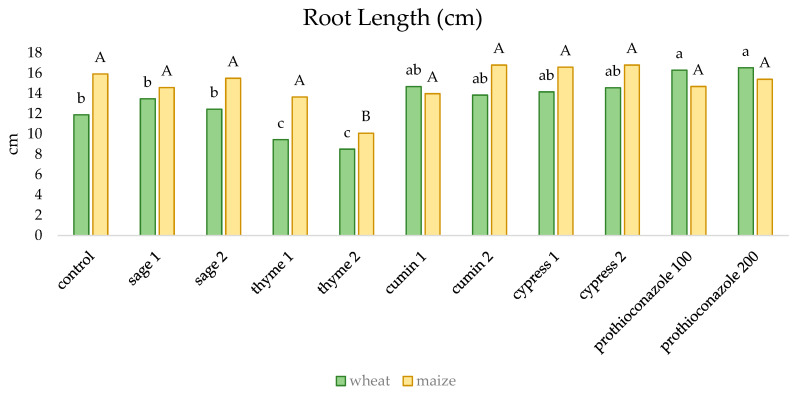
The effect of essential oils and prothioconazole on the length of winter wheat and maize roots (numerical values given in the names of the substances correspond to doses (mL) per 200 mL of water or 100 kg of grain). Different letters indicate statistics different mean HSD (0.05): wheat = 1.899 (lower-case letters), SD = 1.32; maize = 2.313 (capital letters), SD = 1.60.

Individual substances had different effects on the root length of winter wheat and maize (Figure 3). In the case of corn, significantly shorter shoots were observed only when thyme essential oil was used; in the remaining combinations the differences were not statistically significant. The longest winter wheat roots were recorded for the combination in which a.i. prothioconazole was used, and the shortest were recorded for grains soaked in thyme EO, followed by sage EO and the control.

**Figure 4:**
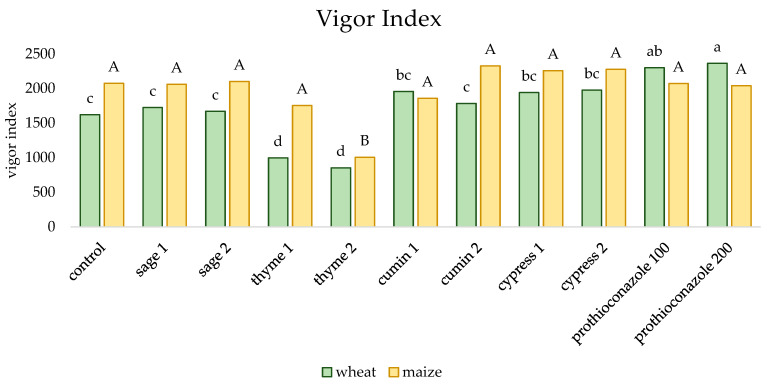
The effect of essential oils and prothioconazole on the vigor index of winter wheat and maize (numerical values given in the names of the substances correspond to doses (mL) per 200 mL of water or 100 kg of grain). Different letters indicate statistics different mean HSD (0.05): wheat = 281.775 (lower-case letters), SD = 195.12; maize = 353.654 (capital letters), SD = 244.90.

In the case of maize, no differences in seedling vigor were found for most combinations; a statistically significant decrease in the value of this parameter was observed only for the combination in which thyme EO was used (Figure 4). The best vigor of winter wheat seedlings was recorded for both doses of a.i. prothioconazole, and the lowest value of this parameter concerned grains soaked in thyme essential oil.

**Figure 5:**
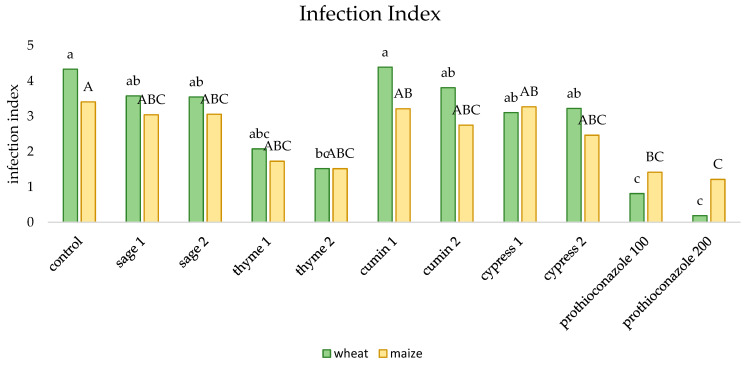
The effect of essential oils and prothioconazole on the infection index of winter wheat and maize (numerical values given in the names of the substances correspond to doses (mL) per 200 mL of water or 100 kg of grain). Different letters indicate statistics different mean HSD (0.05): wheat = 1.547 (lower-case letters), SD = 1.07; maize = 1.163 (capital letters), SD = 0.81.

The lowest level of the infection of corn and winter wheat seedlings was observed for both doses of a.i. prothioconazole, followed by the application of thyme essential oil (Figure 5). The highest level of infection was recorded for the control, and in the case of wheat also for the lower dose of cumin EO. All substances contributed to a decrease in the infection of corn seedlings.

## 3. Discussion

Trans-Thujanol is an important component of thyme essential oil [53]. This substance may have antifungal properties [54]. The composition of sage essential oil is dominated by a-Thujone [55]. Thujone is a substance with antifungal and antibacterial properties [56]. One of the main components of cumin essential oil is ß-Pinene [57]. This substance has antimicrobial properties [58]. Cypress essential oil contains the most monoterpene hydrocarbons α-Pinene [59]. This substance has, among others, antimicrobial and antioxidant properties [60]. The dominance of the described substances was also found in the case of essential oils used in this experiment.

In the described experiment, thyme essential oil had a negative effect on the germination of grains and the development of winter wheat seedlings and, when used at a higher dose, on the growth parameters of corn, but it had a positive effect on their health. In the experiment described by Anžlovar et al. [61], soaking wheat grains in the discussed oil also contributed to a decrease in both the infection by fungal pathogens and the germination capacity of winter wheat grains. However, they indicated that other methods of applying thyme oil (e.g., fumigation) may reduce the negative impact on germination levels. In this experiment, a lower dose of the oil did not negatively affect the level of corn grain germination. This indicates that an appropriate dose of thyme EO can achieve sufficiently high fungicidal effectiveness while being safe for developing seedlings. Winter wheat had a stronger negative reaction to the mentioned essential oil applied at a lower dose. Individual plants had different sensitivity to essential oils [62]; therefore, the introduction of new preparations based on these substances must be preceded by research on various plant species. The harmful effects of essential oils may result from the fact that some of them affect the permeability of cell membranes. They can also inhibit ATP production, which is harmful, especially during seed germination and seedling growth [63].

Infection with pathogens of the *Fusarium* genus adversely affects the levels of germination and development of cereal seedlings [64,65]. The use of appropriate protection allows one to achieve optimal conditions for plant development. The best growth parameters of winter wheat and maize seedlings were found for the treatment in which prothioconazole was used. These combinations also had the lowest infection rates. However, the applied seed treatments must be appropriately selected because some preparations may contribute to the occurrence of phytotoxic effects [66]. The tested essential oils influenced the health of winter wheat and corn seedlings to varying degrees. In research conducted by Grzanka et al. [67], soaking the seeds in clove essential oil contributed more to inhibiting the infection of seedlings than in the case of using pine essential oil. It is therefore worth examining the possibility of using different essential oils to reduce seedling infections, because they could have varying degrees of effectiveness. Of the essential oils used in the experiment, thyme essential oil contributed the most to this effect. Its efficacy was similar to a.i. prothioconazole. In research conducted by Bot et al. [68], it was shown that the use of essential oil vapors from *Coriandrum sativum*, *Origanum vulgare*, and *Thymus vulgaris* inhibited the growth of fungi of the *Drechslera*, *Alternaria*, and *Fusarium* genera. It also reduced the occurrence of deoxynivalenol and grain germination, which, however, depended on the concentration of oils. It did not contribute to the quality of bread baked from grains treated with the listed substances. Essential oils may therefore potentially be a good solution not only as a replacement for chemical dressings but also to protect infected cereal grains that are used in industry.

In the part of the experiment regarding the influence of essential oils on the development of mycelium of pathogens of the *Fusarium* genus, it was assessed that in most tested combinations in which thyme essential oil was used, the growth of the discussed fungi was completely inhibited. In a study conducted by Faghih-Imani et al. [69], it was found that the use of thyme EO contributes to the inhibition of the mycelium growth of pathogens of the *Fusarium* genus as well as their germination and spore production. Palfi et al. [70] assessed the effect of different essential oils on the growth of *Fusarium oxysporum* mycelium. The experiment showed that thyme essential oil had the best properties in this respect among the dozen or so oils used in the experiment. In the experiment, sage essential oil also showed high efficacy in limiting the development of *Fusarium pathogens*. The conducted research showed that sage essential oil strongly inhibited the development of pathogen mycelium, but the level of this effect was largely dependent on the fungus species. *F. graminearum* was the most susceptible to the effects of the substance, but the growth of all species was noticeably slowed down. Yılar et al. [71] in their research showed that sage essential oil also inhibits the growth of *F. oxysporum* mycelium. Cumin essential oil contributed to the complete inhibition of the mycelium growth of *F. graminearum* and *F. avenaceum*, as well as to a significant reduction in the growth of the other two species. Kedia et al. [72] showed that this oil also inhibited the development of *Fusarium oxysporum*. Further research into the use of essential oils is recommended because it is a promising alternative to chemical plant-protection products. These substances are safe for the environment and carry a low risk of residues in water and soil [73], which are sought-after features in the search for new plant-protection methods.

## 4. Materials and Methods

The following essential oils were used in the experiment: sage (*Salvia officinalis* L.), cypress (*Cupressus sempervirens* L.), cumin (*Cuminum cyminum* L.), and thyme (*Thymus vulgaris* L.), which came from a commercial source (Etja, Elbląg, Poland). The compositions of essential oils were assessed according to the methodology of Grzanka et al. [74]. The results include the substances that occurred in the largest amounts. The laboratory experiment was carried out with in vitro conditions on Petri dishes with a diameter of 90 mm into which agar-glucose-potato agar (PDA) medium was poured. The control object was medium without the addition of tested substances. After the medium hardened, discs with a diameter of 5 mm, overgrown with the mycelium of the analyzed fungi species, were placed in the central part. The essential oils to be tested (in two doses: 1 mL and 2 mL) were added to 200 mL PDA medium. The reference object was a combination in which the active ingredient, prothioconazole (Promino 300 EC; CAC Chemical GmbH, Hamburg, Germany; prothioconazole—300 g·L^−^^1^), was added to the medium at a dose of 0.33 L·ha^−^^1^ to 0.65 L·ha^−^^1^ corresponding to the minimum and maximum field dose registered in cereal cultivation. The plates were incubated at 20 °C with 80% humidity and 12/12 light periods. The experiment was carried out in 2 series (5 repetitions each). The tested substances were added to sterile medium cooled to 45 °C in such quantities to obtain a concentration of 0.5 and 1% *w*/*w* of the tested active substances. The diameter of the culture in each combination was measured after observation of mycelium overgrowth onto the substrate surface in a given control object or after 3 weeks in the case of slow-growing cultures. The average mycelium growth in millimeters was calculated, and then the percentage of inhibition of the mycelium growth of a given pathogen by the tested objects was calculated using the formula Ow = (K − F/K)·100, where Ow = percent of inhibition of colony growth, K = diameter of colony in control object, and F = diameter of colony in tested object.

The research material consisted of grain of winter wheat (*Triticum aestivum*) variety Banatus and corn (*Zea mays*) variety Benedictio. A total of 300 g of grain was measured and then inoculated with a suspension of mycelium and spores of fungi of the genus *Fusarium* at a concentration of 10 to 6 spores in 1 mL of suspension in specially prepared containers. The seeds were placed on quality filter paper, dimensions 450 **×** 560 mm, basis weight 80 g/m^2^. To prepare the inoculum, isolates of pathogenic fungi were used, obtained by cultivating pure cultures from plant material from wheat and corn, among others such as *Fusarium avenaceum* (Fr.) Sacc., *Fusarium culmorum* (W.G.Sm.) Sacc., *Fusarium graminearum* Schwabe, and *Fusarium fujikuroi* Nirenberg. The fungal isolates used to inoculate the grain came from a collection of isolates created and maintained at the Department of Mycology of the IOR-PIB in Poznań. For the research at this stage, fungal isolates with the highest pathogenicity were selected and multiplied on appropriate media. For pathogenicity tests, 5 isolates of the species *F. culmorum*, *F. avenaceum*, *F. fujikuori*, and *F. graminearum* were selected and multiplied. In laboratory conditions, surface-disinfected grain of winter wheat and corn was inoculated with a suspension with a spore density established for all isolates of 4 × 10^6^ spores in 1 mL^−1^. The grain was placed on filter paper in large Petri dishes, 100 pieces in 4 repetitions for each isolate. The health of the seedlings was assessed after 7 days of incubation by determining the number of coleoptiles with necrotic symptoms. The results were analyzed statistically using the variance method. One isolate from each species was selected for testing, as it caused the significantly highest percentage of infected seedlings in the test.

The rolled towel test (BP method) was performed in four repetitions per combination, each with 25 grains. The essential oils were mixed with ethoxylated rapeseed oil (Rokacet RZ17, PCC group, Brzeg Dolny, Poland) in a ratio of 9:1 to create a uniform oil formulation that could be dissolved in water. The control sample consisted of seeds that had not been soaked in any solution. In subsequent combinations, the seeds were soaked for 8 min in distilled water with the addition of essential oils in various doses—1.1 mL of emulsified oil × 200 mL^−^^1^ of distilled water and 2.2 mL of emulsified oil × 200 mL^−^^1^ (based on 1 mL of pure essential oil × 200 mL^−^^1^ distilled water and 2 mL pure essential oil × 200 mL^−^^1^ distilled water). In all cases, 25 g of winter wheat grains and 25 g of corn grains were soaked in the solutions. In subsequent combinations, prothioconazole (Gamelan 100 FS; Innvigo Sp z o.o., Warsaw, Poland; prothioconazole—100 g × L^−^^1^) was used in doses of 100 and 200 mL × 100 kg^−^^1^ grain. The rolls were placed in a thermostatic cabinet, ensuring constant humidity at a temperature of 21 °C. After 4 days, the germination energy of seeds was determined. After 7 days from the beginning of the research, the length of the shoots and seedling roots and germination capacity of seeds were assessed. Based on the collected results, the vigor index was determined: vigor index (VI) = [seedling length (cm) × germination (%)]. The infection of seedlings by fungal pathogens was visually determined. The infection index was assessed.

$$\mathrm{Infection}\;\mathrm{index}=\frac{\left(n\left(II\right)\;\times \;0.25\right)+n\left(III\right)\;\times \;0.75)+n(IV)}{n(I+II+III+IV)}$$

where I indicates no symptoms; II is less than 50% of seedlings attacked; III is more than 50% of seedlings attacked; and IV is 100% of seedlings attacked.

Results were analyzed with Statistica 13 software (StatSoft Ltd., Kraków, Poland). Analysis of variance (ANOVA) was used to determine significant differences between treatments. Means were separated by protected Tukey’s HSD test at *p* = 0.05.

## 5. Conclusions

The formulation of essential oils described in the experiment allowed the creation of a liquid that effectively limited the infection of winter wheat and corn seedlings, but the level of effectiveness depended on the type of essential oil. The level of inhibition of mycelial growth of pathogens of the *Fusarium* genus in many variants was at a level equal to that of a synthetic fungicide. Cypress essential oil had the weakest effect in terms of inhibiting fungal growth. In the case of soaking grain in essential oil solutions, thyme essential oil had the best effect in reducing seedling infection, but at the same time it had an adverse effect on the development of seedlings. Essential oils are a promising alternative to synthetic fungicides, but different types need to be tested, both in terms of effectiveness and safety of the crop.

## Figures and Tables

**Table 1:** Main ingredients of the tested essential oils (% in volume *v*/*v*).

Sage		Thyme		Cumin		Cypress	
Chemical Compounds	%	Chemical Compounds	%	Chemical Compounds	%	Chemical Compounds	%
α-Thujone	38.8	Trans-Thujanol	36.1	ß-Pinene	58.3	α-Pinene	44.7
Camphor	28.1	α-Thujene	12.4	Myrcene	26.6	δ-3-Carene	22.8
α-Pinene	6.2	Linalool	10.3	Cuminal	6.8	Limonene	6.4
β-Thujone	5.2	Terpinen-4-ol	14.1				
Camphene	3.2	Cis-Thujanol	5.9				

**Table 2:** The influence of selected essential oils and prothioconazole on the growth of mycelium of pathogens of the genus *Fusarium*.

Treatment		Dose per 200 L of Water	*F. culmorum*	*F. graminearum*	*F. fujikuori*	*F. avenaceum*
No.	Name		Surface of the Mycelium (mm)			
1	control	-	90.0 a	90.0 a	90.0 a	90.0 a
2	sage	1%	10.3 d	0.0 d	26.3 d	10.8 d
3	sage	2%	7.0 de	0.0 d	7.8 e	4.0 fg
4	thyme	1%	0.0 e	0.0 d	0.0 f	0.0 g
5	thyme	2%	0.0 e	0.0 d	0.0 f	0.0 g
6	cumin	1%	1.8 e	0.0 d	2.8 f	0.0 g
7	cumin	2%	4.3 de	0.0 d	0.0 f	0.0 g
8	cypress	1%	69.3 b	69.8 b	76.8 b	81.3 b
9	cypress	2%	40.0 c	34.3 c	55.3 c	51.0 c
10	prothioconazole	0.33 l	7.8 de	8.3 d	6.3 e	8.3 de
11	prothioconazole	0.65 l	0.0 e	6.0 d	0.0 f	6.0 ef
HSD (0.05)			5.64	6.90	3.09	3.37

Different letters a–g indicate statistically different mean values (α = 0.05). % in volume *w*/*w*, l in volume *w*/*w*.

**Table 3:** The effect of essential oils and synthetic fungicide on the energy and germination capacity of winter wheat and maize grain.

Treatment		Dose per 200 mL of Water or 100 kg of Grain (mL)	Winter Wheat		Maize	
No.	Name		Germination Energy (%)	Germination Capacity (%)	Germination Energy (%)	Germination Capacity (%)
1	control	-	92.0 a	96.0 a	94.0 a	94.0 a
2	sage	1	91.0 a	93.0 a	95.0 a	97.0 a
3	sage	2	96.0 a	96.0 a	94.0 a	96.0 a
4	thyme	1	67.0 b	78.0 b	94.0 a	94.0 a
5	thyme	2	47.0 c	72.0 b	64.0 b	68.0 b
6	cumin	1	96.0 a	96.0 a	94.0 a	94.0 a
7	cumin	2	94.0 a	94.0 a	98.0 a	98.0 a
8	cypress	1	95.0 a	95.0 a	97.0 a	97.0 a
9	cypress	2	94.0 a	96.0 a	95.0 a	97.0 a
10	prothioconazole	100	98.0 a	98.0 a	97.0 a	98.0 a
11	prothioconazole	200	99.0 a	99.0 a	96.0 a	96.0 a
HSD (0.05)			8.49	8.75	8.95	10.25
SD			5.88	6.06	6.20	7.10

Different letters a–c indicate statistically different mean values (α = 0.05).

## Data Availability

The source data is stored by the authors and will be available for readers if necessary.
